# A novel immune-related radioresistant lncRNAs signature based model for risk stratification and prognosis prediction in esophageal squamous cell carcinoma

**DOI:** 10.3389/fgene.2022.921902

**Published:** 2022-09-06

**Authors:** Jianqing Zheng, Xiaohui Chen, Bifen Huang, Jiancheng Li

**Affiliations:** ^1^ Clinical Oncology School of Fujian Medical University, Fujian Cancer Hospital, Fuzhou, Fujian, China; ^2^ Department of Radiation Oncology, The Second Affiliated Hospital of Fujian Medical University, Quanzhou, Fujian, China; ^3^ The Graduate School of Fujian Medical University, Fuzhou, Fujian, China; ^4^ Department of Thoracic Surgery, Clinical Oncology School of Fujian Medical University, Fujian Cancer Hospital, Fuzhou, Fujian, China; ^5^ Department of Obstetrics and Gynecology, Quanzhou Medical College People’s Hospital Affiliated, Fuzhou, Fujian, China; ^6^ Department of Radiation Oncology, Clinical Oncology School of Fujian Medical University, Fujian Cancer Hospital, Fuzhou, Fujian, China

**Keywords:** radioresistance, esophageal squamous cell carcinoma, lncRNA, prognostic model, bioinformatics

## Abstract

**Background and purpose:** Radioresistance remains a major reason of radiotherapeutic failure in esophageal squamous cell carcinoma (ESCC). Our study is to screen the immune-related long non-coding RNA (ir-lncRNAs) of radiation-resistant ESCC (rr-ESCC) *via* Gene Expression Omnibus (GEO) database and to construct a prognostic risk model.

**Methods:** Microarray data (GSE45670) related to radioresistance of ESCC was downloaded from GEO. Based on pathologic responses after chemoradiotherapy, patients were divided into a non-responder (17 samples) and responder group (11 samples), and the difference in expression profiles of ir-lncRNAs were compared therein. Ir-lncRNA pairs were constructed for the differentially expressed lncRNAs as prognostic variables, and the microarray dataset (GSE53625) was downloaded from GEO to verify the effect of ir-lncRNA pairs on the long-term survival of ESCC. After modelling, patients are divided into high- and low-risk groups according to prognostic risk scores, and the outcomes were compared within groups based on the COX proportional hazards model. The different expression of ir-lncRNAs were validated using ECA 109 and ECA 109R cell lines *via* RT-qPCR.

**Results:** 26 ir-lncRNA genes were screened in the GSE45670 dataset with differential expression, and 180 ir-lncRNA pairs were constructed. After matching with ir-lncRNA pairs constructed by GSE53625, six ir-lncRNA pairs had a significant impact on the prognosis of ESCC from univariate analysis model, of which three ir-lncRNA pairs were significantly associated with prognosis in multivariate COX analysis. These three lncRNA pairs were used as prognostic indicators to construct a prognostic risk model, and the predicted risk scores were calculated. With a median value of 2.371, the patients were divided into two groups. The overall survival (OS) in the high-risk group was significantly worse than that in the low-risk group (*p* < 0.001). The 1-, 2-, and 3-year prediction performance of this risk-model was 0.666, 0.702, and 0.686, respectively. In the validation setting, three ir-lncRNAs were significantly up-regulated, while two ir-lncRNAs were obviouly down-regulated in the responder group.

**Conclusion:** Ir-lncRNAs may be involved in the biological regulation of radioresistance in patients with ESCC; and the prognostic risk-model, established by three ir-lncRNAs pairs has important clinical value in predicting the prognosis of patients with rr-ESCC.

## Introduction

Esophageal cancer (EPC) is one of the most lethal tumors in China and worldwide (Sung et al., 2021). Esophageal squamous cell carcinoma (ESCC) is the major pathologic type in Chinese population, accounting for more than 90% cases, and more than 45%–60% of them were diagnosed at an advanced stage (Yang et al., 2018). In general, the prognosis of EPC is very poor, the cancer-specific mortality rate ranks fourth, and the 5-year overall survival rate is less than 30% (Chen et al., 2016; Zeng et al., 2018). Radiotherapy is one of the main treatments for EPC, especially in patients at an advanced stage. However, after radiotherapy with/without chemotherapy, the total objective response rate (ORR) is only 60%–76%, and radiotherapy resistance is an important reason for the local failure of radiotherapy for EPC (Chen et al., 2021). Although multiple factors may affect the radiosensitivity, the specific mechanisms of long non-coding RNA (lncRNAs) in radioresistance are still worth exploring. The outcomes of radiotherapy are heterogeneous, and no clinical or pathological method could predict tumor response of radiotherapy.

LncRNA referred to a type of RNAs whose length is greater than 200 bp and does not encode or translate proteins after transcription. LncRNAs are involved in various physiological and pathological processes, such as the cellular replication, transcription, translation and so on. In the process of gene expression and transcription, about 70% of human genes are regulated by lncRNAs (Batista and Chang, 2013; Shi et al., 2013). It’s reported that lncRNAs are widely involved in the biological processes of cancer, including tumor radioresistance (Li and Chen, 2013). Many studies believed that the prognosis of tumors is closely related to the tumor microenvironment (TME), and lncRNAs may participate in the regulation of TME through molecular biological functions such as chromatin modification, transcription, and post-transcriptional processing (Sounni and Noel, 2013; Singh et al., 2016). In addition, some lncRNAs are also involved in the regulation of immune function in the TME, where may involve various types of cells and cytokines. Such lncRNAs are often referred to as immune-related lncRNAs (ir-lncRNAs) (Bremnes et al., 2016; Chen M. M. et al., 2020). In order to explore whether ir-lncRNAs are involved in the biological process of radio-resistance in patients with ESCC, our study analyzed the differences in the expression of ir-lncRNAs between ESCC-patients with radioresistance and complete tumor remission based on gene expression profiling data in the Gene Expression Omnibus (GEO) database. Ir-lncRNA pairs, which had high correlation with immune genes, were selected to establish a prognostic risk-model to explore the value of ir-lncRNAs in predicting the prognosis of ESCC patients with radioresistance, and further to find potential biomarkers of radioresistance.

## Materials and methods

### Dataset collection and preparation

Using “esophageal cancer, esophagus cancer, and radioresistance” as keywords, mRNA and lncRNA expression profiling data related to radioresistance of esophageal cancer was searched in the GEO database (https://www.ncbi.nlm.nih.gov/). Two public esophageal cancer microarray profiling datasets (GSE45670 and GSE53625) were downloaded from GEO and were finally selected for data mining. The GSE45670 microarray profiling datasets were provided by Wen et al., where gene expression analyses were performed on pretreatment cancer biopsies from 28 ESCCs who received neoadjuvant chemoradiotherapy (CRT) and surgery and 10 normal esophageal epithelia using Affymetrix U133 Plus 2.0 arrays (Wen et al., 2014). After preoperative chemoradiotherapy among 28 ESCCs, complete remission of tumor occurred in 11 patients, who were divided into responder group, while no obvious tumor regression occurred in the other 17 patients, who were considered to be radioresistant and divided into non-responder group. The average age of the patients in the non-responder group was (55.65 ± 5.53) years old, of whom 16 patients were male (accounting for 94.11%); four patients were in T2N1M0 stage, and 13 patients were in T3N1M0 stage; while average age of the patients in the responder group was (57.45 ± 6.80) years old, of whom nine patients were male (accounted for 81.82%); four patients were in T2N1M0 stage and seven patients were in T3N1M0 stage.

The GSE53625 microarray profiling datasets were provided by Li et al.(Shi et al., 2016; Li et al., 2017; Liu et al., 2020), including 179 patients with esophageal cancer. The profiling datasets were derived from cancer tissue and adjacent normal tissue. The dataset contained detailed clinical information of 179 ESCC patients that can be used for prognostic validation analysis. Both datasets were annotated with the platform files provided by GEO to obtain Ensemble ID, and then were annotated with the “org.Hs.eg.db” package to obtain gene symbols, thus ensuring that the two datasets have similar annotation conditions.

### Screening, differential expression analysis and matched pairs of ir-lncRNAs

The immune gene list was download from the ImmuPORT database (https://www.immport.org/). Subsequently, the subsets of immune genes or lncRNAs were extracted from the GSE45670 dataset and GSE53625, respectively. The correlation test was performed on the immune gene expression matrix and the lncRNA expression matrix, and the related lncRNAs were screened as ir-lncRNAs. The screening criteria were: correlation coefficient *ρ* ≥ 0.4 or *ρ* ≤ −0.4 and *p* ≤ 0.0001. Differential expression analysis (DEA) was performed on eligible ir-lncRNAs *via* the “limma” and “SVA” packages, and the expression difference was defined as: the absolute value of the log2 value (fold change) of the difference fold was greater than (mean ± 2 times the standard deviation of expression); *p* value ≤ 0.05. The differentially expressed lncRNAs were paired for each other. Taking the LINC01121|FAM167A-AS1 gene as an example, if the expression of LINC01121 gene was greater than that of FAM167A-AS1 gene, the matched index was recorded as 1, otherwise as 0. The ir-lncRNA pairs were introduced as independent variables into the COX prediction model to establish a prognostic risk-model.

### Construction of the predictive model

The clinical survival data of ESCC samples were extracted from the GSE53625 dataset, and the ir-lncRNA pairs at the common intersection between the GSE45670 dataset and the GSE53625 dataset were considered as components of the survival data.

Univariate and multivariate Cox regression survival analyses were performed using the “survival” package in R 4.1.2 software to screen ir-lncRNAs with significant impact on prognosis. The least absolute shrinkage and selection operator (LASSO) regression analysis was carried out to narrow down the prognostically significant ir-lncRNA pairs. An ir-lncRNAs pairs-based risk prediction model was established with ir-lncRNAs of statistically significant differences in both univariate and multivariate Cox regression survival analysis.

The model building formula is (Huang et al., 2021):

$$\text{Riskscore}=\overset{n}{\underset{i=1}{\sum }}\left(lncRNApairsi\times coefi\right).$$

Where *n* is the counts of ir-lncRNA pairs, and lncRNApairsi and coefi represent the related matched results (values of 1 or 0) and coefficients of modeled ir-lncRNA pairs, respectively.

After the riskscores of all the included samples in GSE53625 dataset were calculated, the samples were divided into low-risk group and high-risk group according to the median value of riskscores. Furthermore, the Kaplan-Meier survival curve was applied to analyze the difference of survival prognosis between the high- and low-risk groups with Survival package. The ROC curves of 1-, 2-, and 3-year of overall survival were plotted with ROC package. Univariate and multivariate Cox regression survival analyses were performed with ESCC baseline data and riskscores *via* the “survival” package in R 4.1.2 software, respectively, to explore the independent prognostic factors of ESCC.

### Differential analysis of riskscore for different clinical characteristics

The clinical characteristics such as gender, age, tumor TNM stage, etc. of ESCC samples were extracted from GSE53625 dataset and combined with the corresponding risk scores. Wilcoxon rank sum test was performed *via* the “ggpubr” package and the boxplots was drawn to show the correlations between risk scores and clinical characters.

### Differential expression analysis of immune-related genes in different radiosensitivity groups

Based on those ir-lncRNAs screened from the GSE53625 dataset with prediction model, the immune-related genes in the GSE45670 dataset were reversely extracted, and the differential expression analysis of immune-related genes were compared between the non-responder group and the responder group. A heat map and a volcano plot were drawn.

### Gene ontology, Kyoto encyclopedia of genes and genomes functional enrichment analysis

For the purpose of exploring the molecular mechanism of ir-lncRNAs and ir-genes related to radio-resistance in ESCC, we implemented gene ontology (GO) and Kyoto encyclopedia of genes and genomes (KEGG) functional enrichment analysis *via* the “clusterProfiler” R package. In these analyses, a *p* value < 0.05 was considered statistically significant. GO enrichment analysis was composed of cellular components (CC), molecular functions (MF), and biological processes (BP), which showed the biological functions of genes at different levels, respectively. KEGG pathway enrichment analysis was used to evaluate the enrichment degree of genes in different pathways. The function of selected ir-genes whose expression were significantly different between the non-responder group and the responder group could indirectly speculate on the biological roles and mechanisms of immune-related lncRNAs.

### lncRNAs isolation, cDNA synthesis, and RT-qPCR

Cellular total RNAs were isolated via TRIzol reagent (Thermo, United States) from ECA-109 cell lines and ECA-109R cell lines, where ECA-109R were identified as non-responder esophageal cancer and ECA-109 was identified as responder esophageal cancer. To increase the specificity of the real-time quantitative PCR (RT-qPCR), first-strand cDNA was synthesized from 1 mg total RNA *via* RevertAid First Strand cDNA Synthesis Kit (Invitrogen, China). The relative expression level of the target lncRNAs was detected by three-step RT-qPCR *via* the Fast SYBR Green Master Mix (Applied Biosystems Inc., CA, United States). The cycling conditions were 5 min of pre-degeneration at 95°C and 30 s of denaturation (polymerase activation) at 95°C followed by 40 cycles of annealing of primers at 95°C for 5 s and extension of primers at 60°C for 30 s. GAPDH was applied as an internal reference control. The relative expression level was calculated by the relative quantification 2^−ΔΔCT^ method. The experiment was repeated three times and the primer sequences were listed in Supplementary Table S1.

### Statistical analysis

All statistical analyses were performed *via* R software (version 4.1.2). The R packages involved the “Biobase” package, the “GEOquery” package, the “survival” package, the “ggpubr” package, and so on. The one-way ANOVA, Student’s *t*-test (2-tailed) methods or Wilcoxon rank sum test were applied to assess the statistical significance. Quantitative data were shown as the mean ± SE. A *p* value < 0.05 was considered statistically significant.

## Results

### Screening results of ir-lncRNAs in GSE45670 and GSE53625 datasets

**FIGURE 1:**
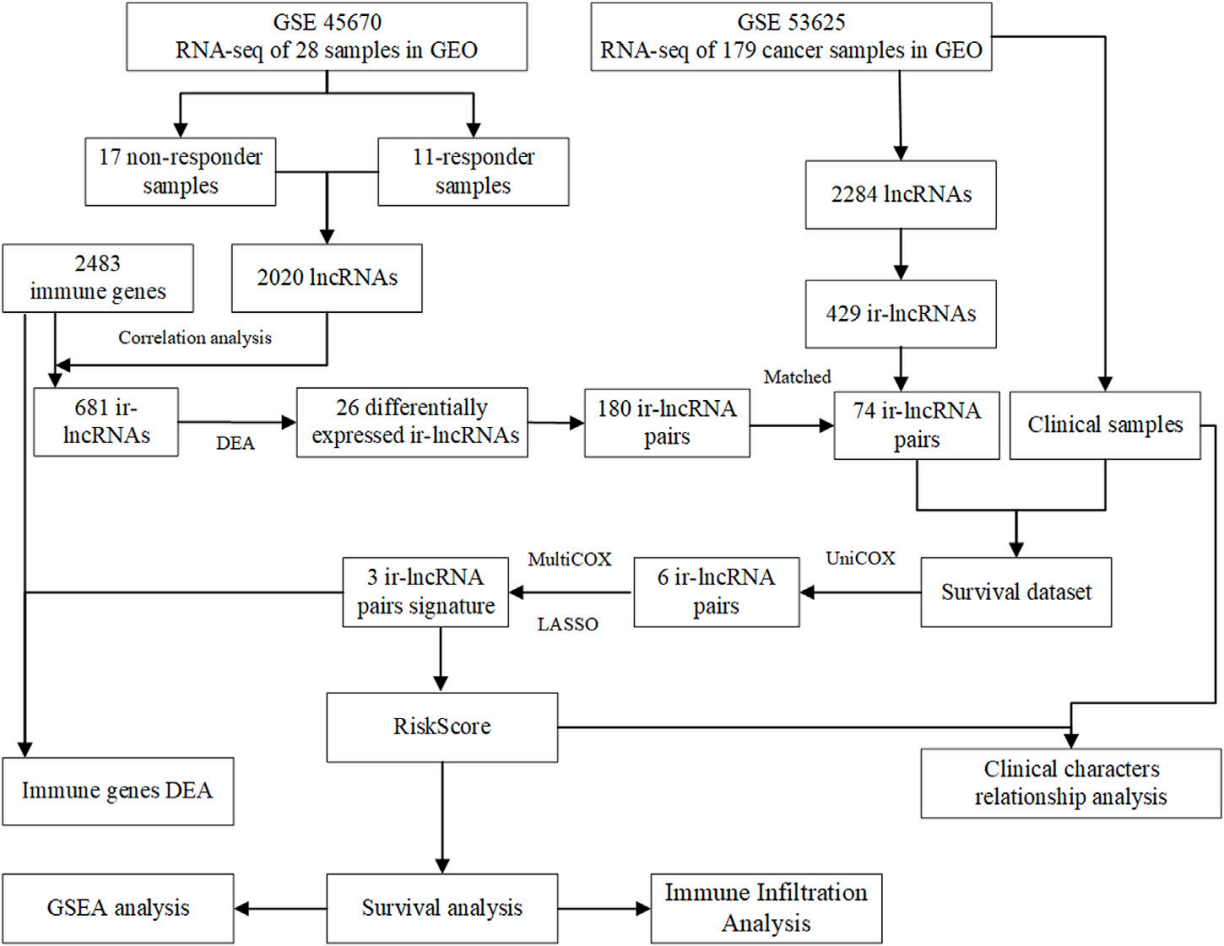
Flow chart of data collection and analysis.

**FIGURE 2:**
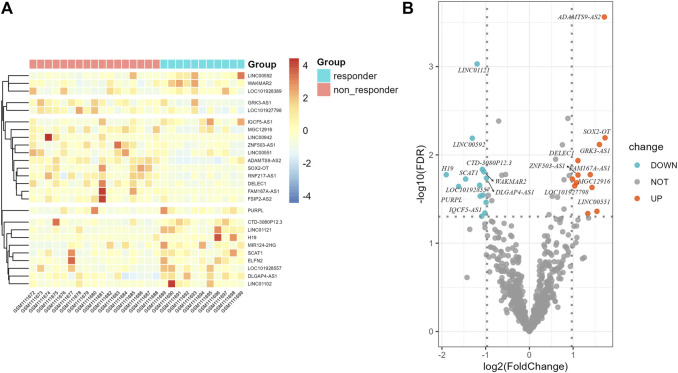
Differential expression results of immune-related lncRNAs in GSE45670 cohort. **(A)** Heatmap, **(B)** Volcano map.

Flow chart of data collection and analysis is shown in Figure 1. According to the screening criteria, a total of 681 ir-lncRNAs were screened in the GSE45670 dataset. Further DEA showed that 12 ir-lncRNAs were up-regulated and 14 ir-lncRNAs were down-regulated in the non-responder group (*p* < 0.05). The results are indicated in Table 1 and Figure 2.

**TABLE 1:** Differential expression results of immune-related lncRNAs in GSE45670.

Genes	log FC	Ave Expr	*t*	*p* value	adj *p* value	Change
LINC01121	−1.196	3.898	−3.679	0.001	0.318	Down
LINC00592	−1.306	7.669	−2.93	0.006	0.589	Down
CTD-3080P12.3	−1.076	3.852	−2.595	0.015	0.589	Down
WAKMAR2	−1.041	7.665	−2.569	0.016	0.589	Down
H19	−1.902	8.538	−2.536	0.017	0.589	Down
DLGAP4-AS1	−0.982	5.319	−2.497	0.018	0.589	Down
SCAT1	−1.459	5.612	−2.487	0.019	0.589	Down
LOC101928557	−1.139	4.483	−2.417	0.022	0.589	Down
PURPL	−1.619	3.829	−2.403	0.023	0.589	Down
IQCF5-AS1	−1.061	3.662	−2.302	0.029	0.641	Down
ELFN2	−1.126	6.26	−2.291	0.029	0.641	Down
MIR124-2HG	−0.991	4.119	−2.217	0.034	0.69	Down
LINC01102	−1.018	2.774	−2.088	0.046	0.825	Down
LOC101928389	−1.09	3.768	−2.048	0.05	0.825	Down
ADAMTS9-AS2	1.714	5.225	4.129	0	0.187	Up
SOX2-OT	1.731	5.417	2.935	0.006	0.589	Up
GRK3-AS1	1.601	3.371	2.864	0.008	0.589	Up
DELEC1	1.11	3.089	2.691	0.012	0.589	Up
FAM167A-AS1	1.391	3.184	2.536	0.017	0.589	Up
ZNF503-AS1	1.112	7.336	2.532	0.017	0.589	Up
RNF217-AS1	0.984	5.366	2.486	0.019	0.589	Up
MGC12916	1.079	4.97	2.446	0.021	0.589	Up
LOC101927798	1.036	5.289	2.409	0.022	0.589	Up
LINC00551	1.432	6.052	2.391	0.023	0.589	Up
LINC00942	1.545	6.705	2.109	0.044	0.824	Up
FSIP2-AS2	1.335	5.226	2.081	0.046	0.825	Up

The 26 differentially expressed ir-lncRNAs genes constituted 325 ir-lncRNA pairs, of which 180 ir-lncRNA pairs were extracted according to the matching rate [pairRaito in the range of (0.2–0.8)], and the data are shown in Supplementary Table S2. Subsequently, we used the same method to construct the ir-lncRNA pairs in the GSE53625 dataset. Finally, 74 ir-lncRNA pairs successfully matched between the GSE45670 and the GSE53625 dataset after intersection. The detailed information of the 74 ir-lncRNA pairs is shown in Supplementary Table S3.

### Clinical characteristics of esophageal squamous cell carcinoma samples in the GSE53625 dataset

The detailed clinical characteristics of GSE53625 ESCC are shown in Table 2.

**TABLE 2:** Clinical characteristics of esophageal squamous cell carcinoma samples in GSE53625.

Items	Overall (*n* = 179, %)	Items	Overall (*n* = 179, %)
Gender		Age	
Female	33 (18.44)	Mean (SD)	53.9 (9.03)
Male	146 (81.56)	Median (min, max)	59.7 (36.0,82.0)
Tobacco		Alcohol	
Yes	114 (63.69)	yes	106 (59.22)
T stage		N stage	
T1	12 (6.7)	N0	83 (46.37)
T2	27 (15.08)	N1	62 (34.64)
T3	110 (61.45)	N2	22 (12.29)
T4	30 (16.76)	N3	12 (6.7)
TNM stage		Tumor grade	
Stage I	10 (5.59)	Poorly	49 (27.37)
Stage II	77 (43.02)	Moderately	98 (54.75)
Stage III	92 (51.4)	Well	32 (17.88)

### Univariate and multivariate COX regression analysis of differential ir-lncRNAs

**FIGURE 3:**
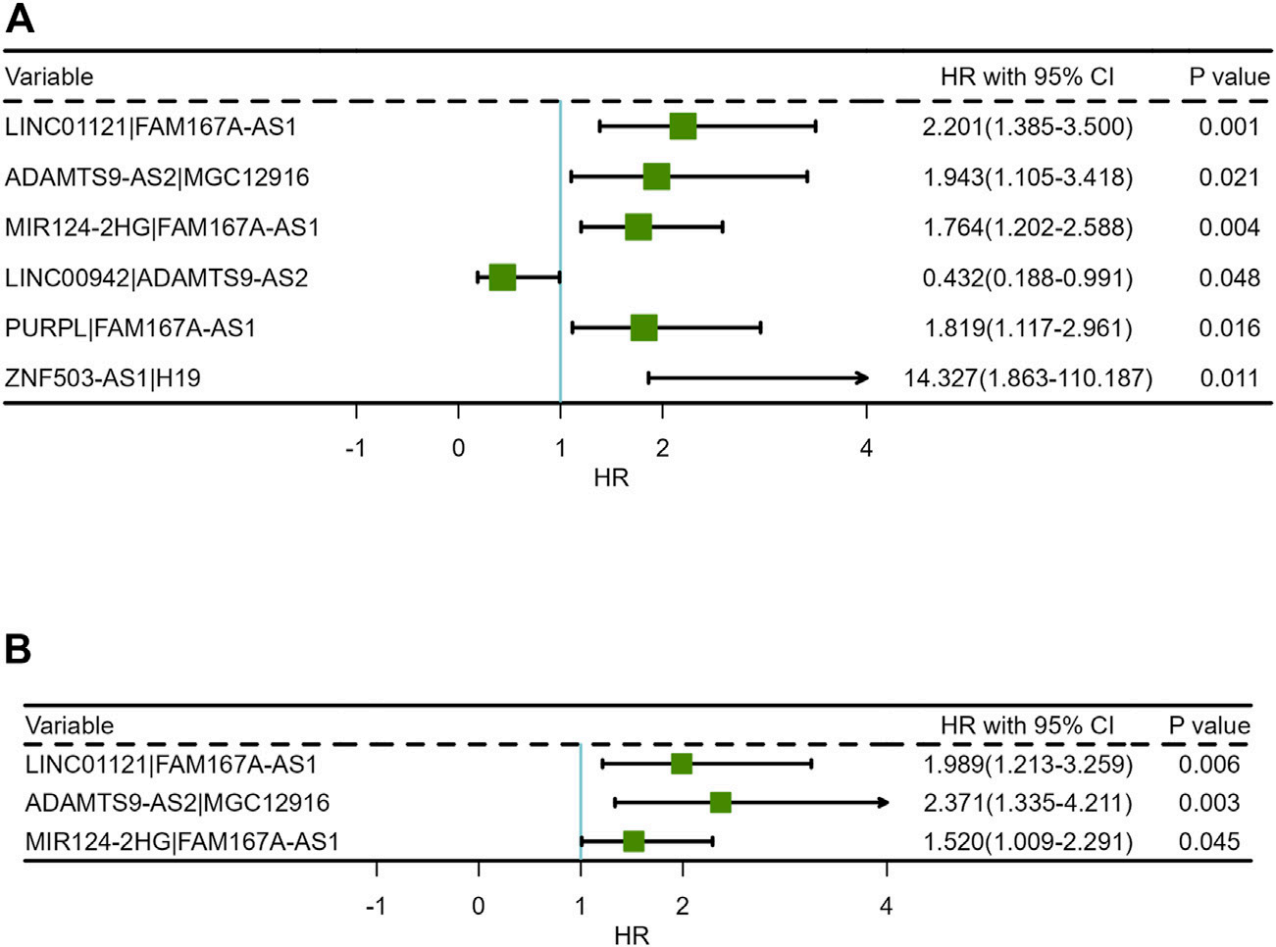
Forest plots of Cox regression analysis for ir-lncRNA pairs: **(A)** Univariate Cox regression analysis, **(B)** Multivariate Cox regression analysis.

**FIGURE 4:**
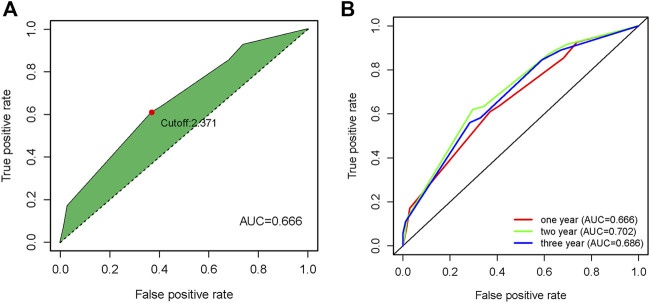
Time ROC curves on overall survival prediction in the GSE53625 cohort **(A)** Time ROC curves with cutoff value, **(B)** Time ROC curves at one year, two year and three year.

The results of univariate COX analysis showed that there were six ir-lncRNA pairs of statistical significance associated with survival outcomes (*p* < 0.05). In order to verify the positive variables obtained by univariate COX regression, LASSO regression (Least absolute shrinkage and selection operator) was further used to screen significant ir-lncRNAs as independent variables for multivariate COX analysis. As a result, five ir-lncRNA pairs, that is, LINC01121|FAM167A-AS1, ADAMTS9-AS2|MGC12916, MIR124-2HG|FAM167A-AS1, LINC00942|ADAMTS9-AS2, and PURPL|FAM167A-AS1, were the most powerful explanatory set of ir-lncRNAs as potential independent variables in multivariate COX model. A further multivariate COX regression was performed, and the results showed that three ir-lncRNA pairs were statistically significant (*p* < 0.05), namely LINC01121|FAM167A-AS1, ADAMTS9-AS2|MGC12916, and MIR124-2HG|FAM167A-AS1. The above analysis revealed that the three ir-lncRNA pairs could serve as independent prognostic factors for ESCC. Since the hazard ratios of the three ir-lncRNAs were all greater then 1, they were considered to be risk factors for the prognosis of ESCC (as shown in Table 3 and Figure 3). Lasso regression road map and selection map were shown in Supplementary Figures S1, S2. ROC curves were used to assess the predictive ability and accuracy of the predictive models based on the three ir-lncRNA pairs. The results showed that the best cut-off point was 2.371 and the area under the curve (AUC) at 1-, 2-, and 3-year were 0.666, 0.702, and 0.686, respectively, as shown in Figures 4A,B.

**TABLE 3:** Univariate and multivariate COX regression analysis of differential ir-lncRNAs.

Ir-lncRNA pairs	Model	*β*	se	HR	HR.95L	HR.95H	*p*
LINC01121|FAM167A-AS1	Univariate	0.789	0.237	2.201	1.385	3.5	0.001
ADAMTS9-AS2|MGC12916	Univariate	0.664	0.288	1.943	1.105	3.418	0.021
MIR124-2HG|FAM167A-AS1	Univariate	0.567	0.196	1.764	1.202	2.588	0.004
LINC00942|ADAMTS9-AS2	Univariate	−0.84	0.424	0.432	0.188	0.991	0.048
PURPL|FAM167A-AS1	Univariate	0.598	0.249	1.819	1.117	2.961	0.016
ZNF503-AS1|H19	Univariate	2.662	1.041	14.327	1.863	110.187	0.011
LINC01121|FAM167A-AS1	Multivariate	0.687	0.252	1.989	1.213	3.259	0.006
ADAMTS9-AS2|MGC12916	Multivariate	0.863	0.293	2.371	1.335	4.211	0.003
MIR124-2HG|FAM167A-AS1	Multivariate	0.419	0.209	1.52	1.009	2.291	0.045

Note: HR.95L: lower limit of 95% confidence interval; HR.95H: upper limit of 95% confidence interval.

Additionally, Supplementary Figure S3 provides differential distribution of riskscore with different clinical characters.

### Construction of ir-lncRNAs-based risk model and survival analysis of high- and low-risk groups

The model formula obtained from multivariate COX regression analysis was:

Riskscore=0.687×LINC01121|FAM167A−AS1+0.863×ADAMTS9−AS2|MGC12916+0.419×MIR124−2HG|FAM167A−AS1

**FIGURE 5:**
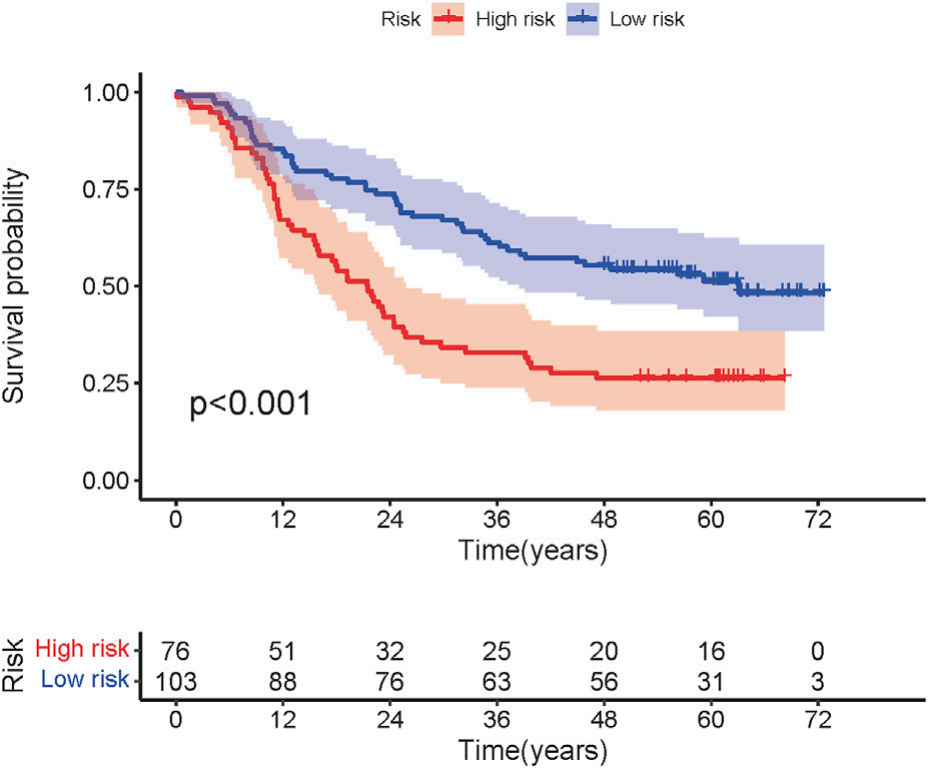
Kaplan-Meier curves for the overall survival of patients in the high- and low-risk group.

The median riskscore was 1.989, and according to the median riskscore, 179 cases were divided into the high- (N = 76) and low-risk (N = 103) groups in the GSE53625 cohort. The Kaplan-Meier survival curves shows that patients in the high-risk group had a worse OS than patients in the low-risk group with more death (*p* < 0.001, Figure 5). The 1-, 3-, and 5-year survival rates in the low-risk group were higher than those in the high-risk group.

### Differential expression analysis of ir-lncRNAs in high- and low-risk groups

**FIGURE 6:**
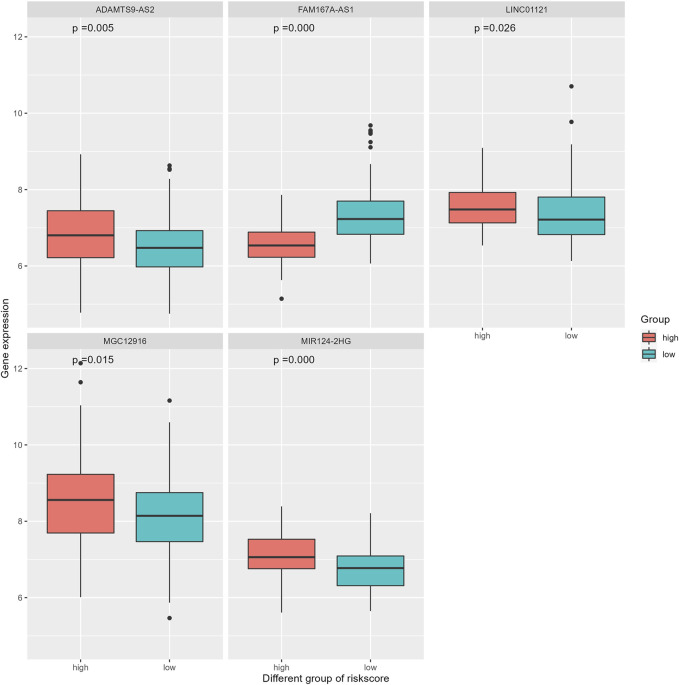
Different expression of five ir-lncRNAs between the low- and the high-risk group.

Based on the aforementioned results, independent sample *t*-test were further applied to assess the expression of ir-lncRNAs in different riskscore groups. The distribution of risk scores is presented in Figure 6. Among the five significant lncRNAs, only FAM167A-AS1 had a decreased expression in the high-risk group, suggesting that FAM167A-AS1 is a protective factor.

### Univariate and multivariate COX regression analysis of clinical characteristics

**FIGURE 7:**
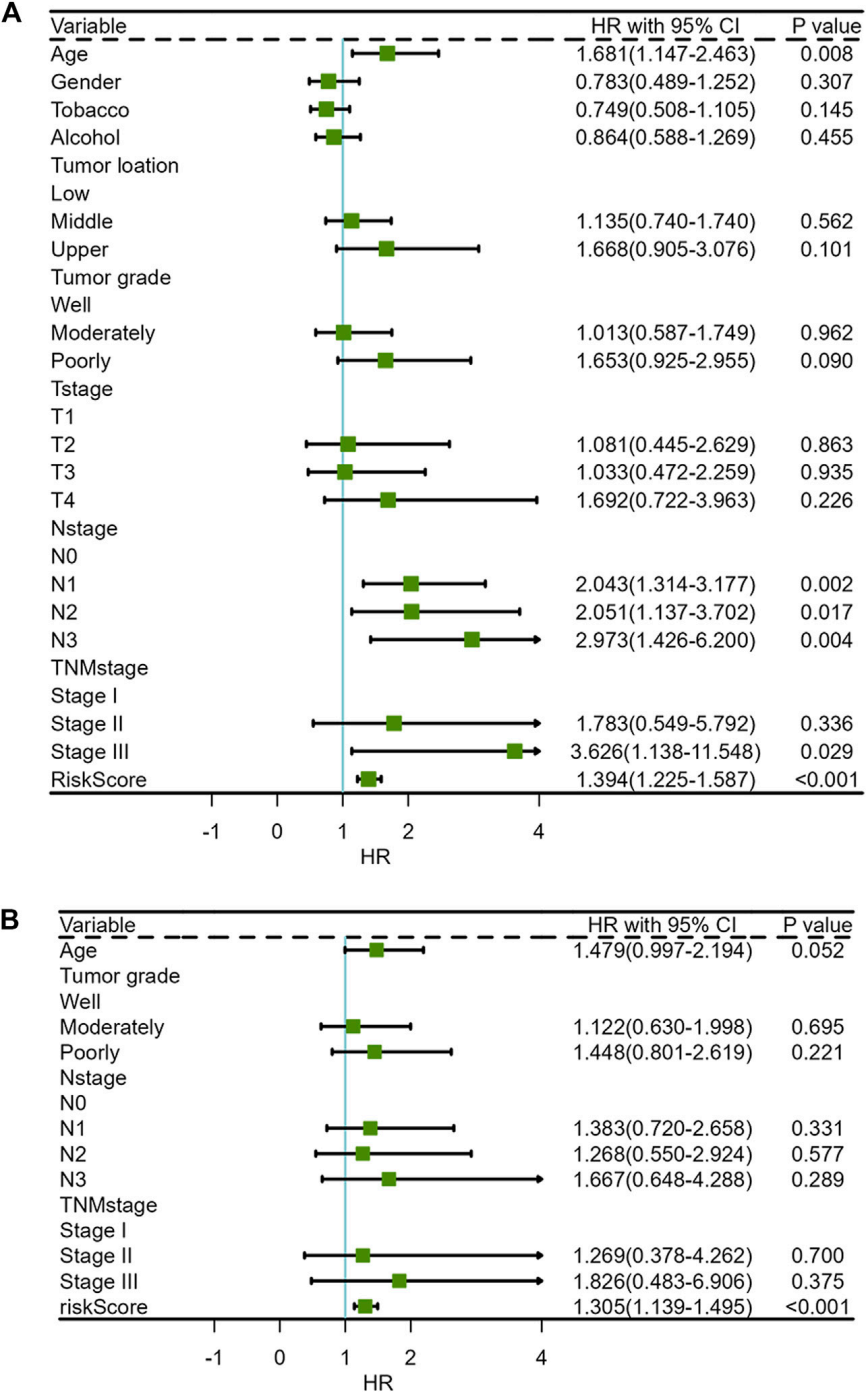
Forest plots of clinical characteristics and risk score for univariate and multivariate COX regression analysis **(A)** Univariate Cox regression analysis, **(B)** Multivariate Cox regression analysis.

The clinical characteristics parameters in Table 2 combined with riskscore were selected to perform univariate and multivariate COX regression analysis. Univariate COX regression analysis demonstrated that age (HR = 1.681, 95% CI = 1.147–2.463, and *p* = 0.008), N-stage (Lymph node staging, N2 vs. N0, HR = 2.051, 95% CI = 1.137–3.702, and *p* = 0.017; N3 vs. N0, HR = 2.973, 95% CI = 1.426–6.200, and *p* = 0.004), TNM-stage (Stage III vs. Stage I, HR = 3.626, 95% CI = 1.138–11.548, and *p* = 0.029) and risk score (HR = 1.394, 95% CI = 1.225–1.587, and *p* < 0.001), which were all negative prognostic factors of OS in the GSE53625 cohort (Supplementary Table S4; Figure 7A). After adjusting for age, tumor grade, N-stage and TNM-stage, multivariate Cox analysis demonstrated that only risk score was a negative prognostic factor of OS (HR = 1.305, 95% CI = 1.139–1.495, and *p* < 0.001) (Supplementary Table S5; Figure 7B).

### CIBERSORT immune infiltration analysis

**FIGURE 8:**
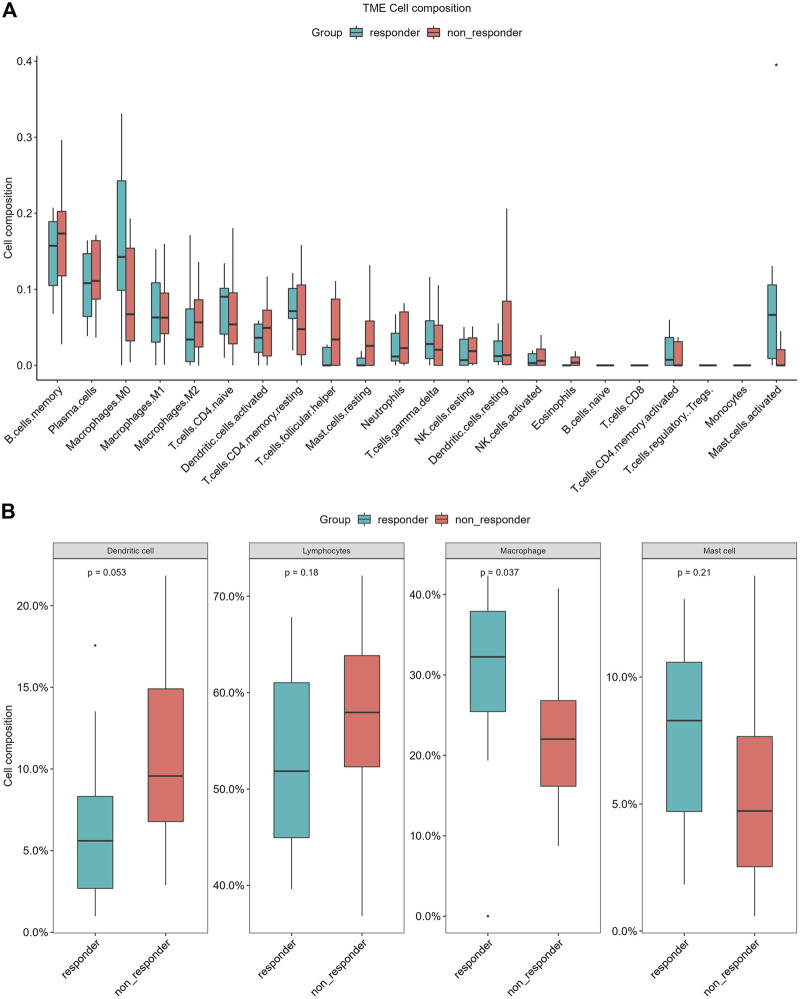
Differences of infiltrating immune cell types between the non-responder group and the responder group of CIBERSORT in GSE45670 cohort. **(A)** 22-classification method, **(B)** 4-classification method.

To explore the difference of tumor immunity landscape between patients in the non-responder group and patients in the responder group, the CIBERSORT algorithm was utilized to evaluate immunity infiltration in the GSE 45670 dataset. The main results are shown in Figures 8A,B. According to the 22-classification method (Newman et al., 2019), the proportions of infiltrating activated mast cells were significantly higher in the responder group (*p* < 0.05). In addition, the proportions of infiltrating macrophages. M0 were also increased in the responder group, although the difference was not statistically significant (*p* > 0.05). The 4-classification method showed that the infiltration level of macrophages in the responder group was significantly higher than that in the non-responder group (*p* < 0.05) (Li B. et al., 2019).

### Differential expression analysis of immune genes

**FIGURE 9:**
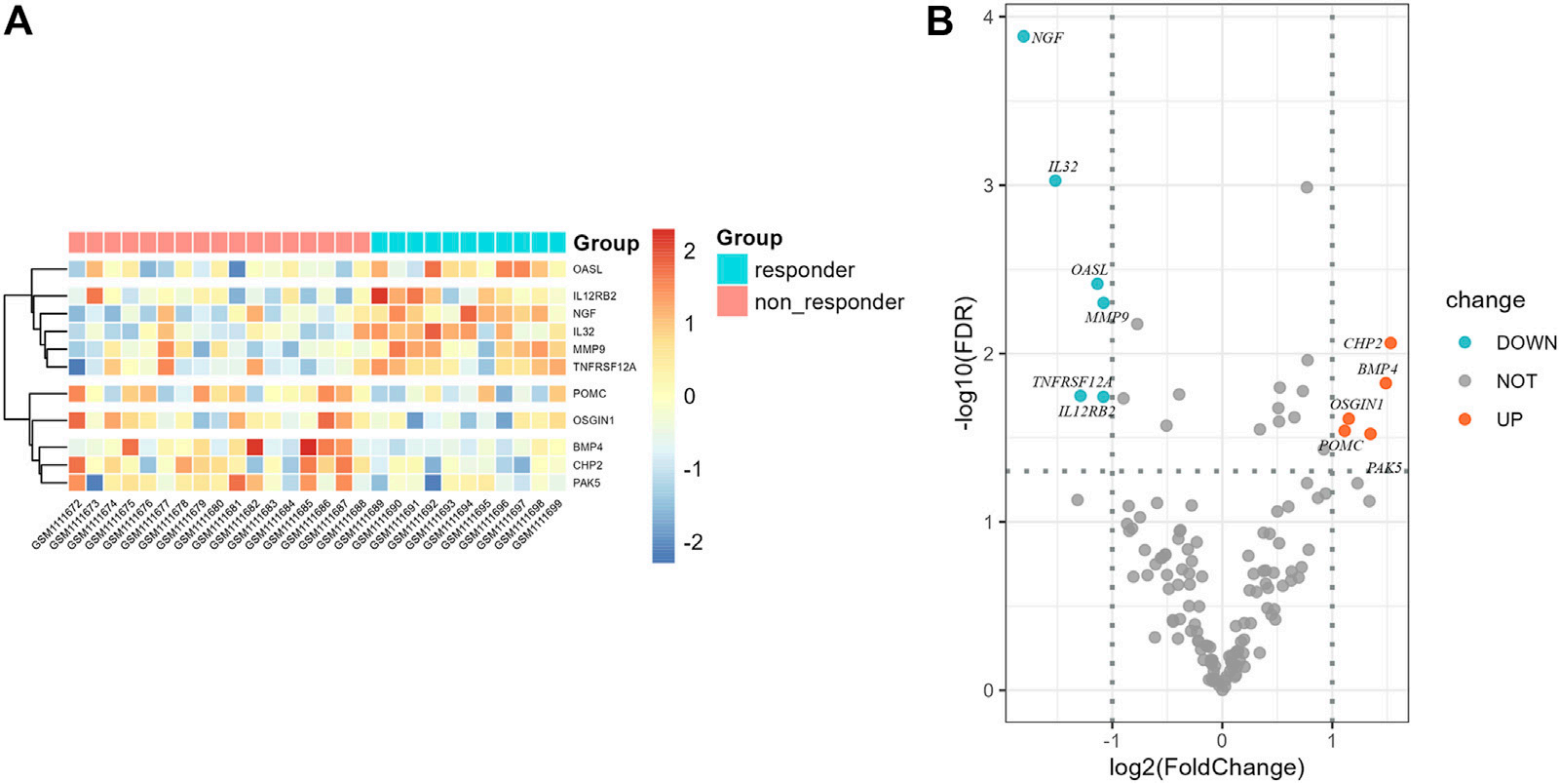
Differential expression results of immune genes in GSE45670 cohort. **(A)** Heatmap, **(B)** Volcano map.

**FIGURE 10:**
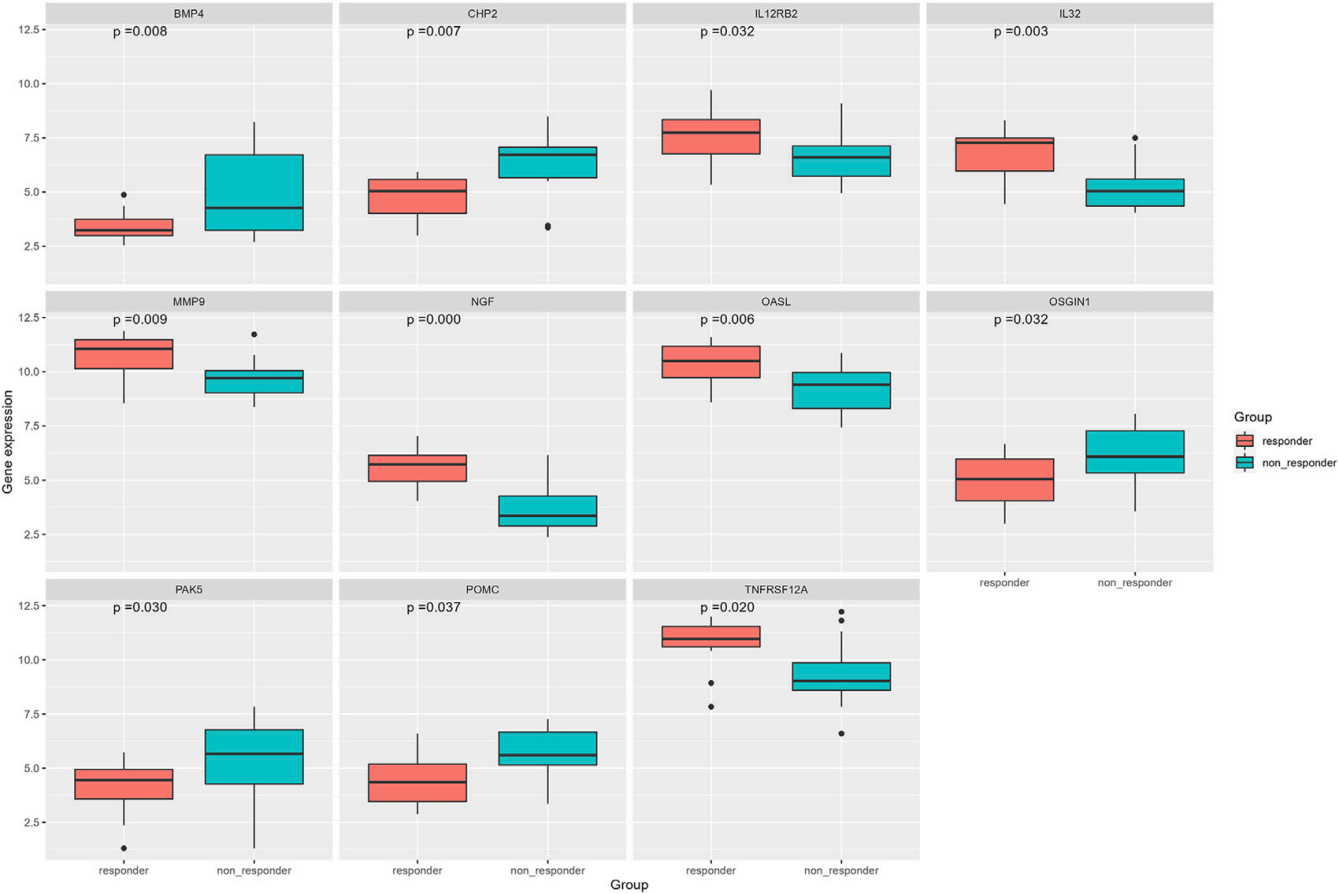
Differential expression analysis of immune genes in GSE45670 cohort.

**FIGURE 11:**
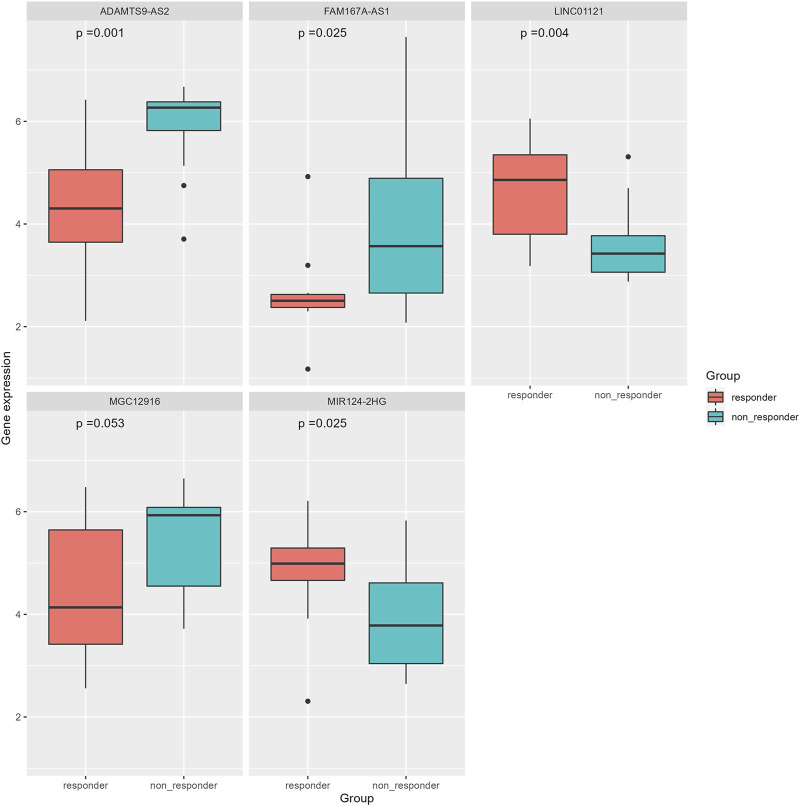
Differential expression analysis of five ir-lncRNAs in GSE45670 cohort.

The correlation coefficient *ρ* = ±0.4 and *p* < 0.05 were used to screen the immune genes related to the above five ir-lncRNAs, and a total of 137 immune genes were screened, of which 11 genes had significant differences in expression levels between the non-responder group and the responder group (*p* < 0.05). Six genes (IL12RB2, IL32, MMP9, NGF, OASL, and TNFRSF12A) were down-regulated and 5 genes (BMP4, CHP2, OSGIN1, PAK5, and POMC) were up-regulated. The distribution of immune genes expression was presented in Figures 9 and 10. Further, we analyzed the expression differences of 5 ir-lncRNAs from GSE45670 cohort. The distribution of ir-lncRNAs expression was presented in Figure 11. Among the above five ir-lncRNAs, ADAMTS9-AS2, and FAM167A-AS1 were up-regulated in the non-responder group, while LINC01121 and MIR124-2HG were down-regulated (*p* < 0.05).

### Functional gene ontology and Kyoto encyclopedia of genes and genomes enrichment analysis of immune genes

**FIGURE 12:**
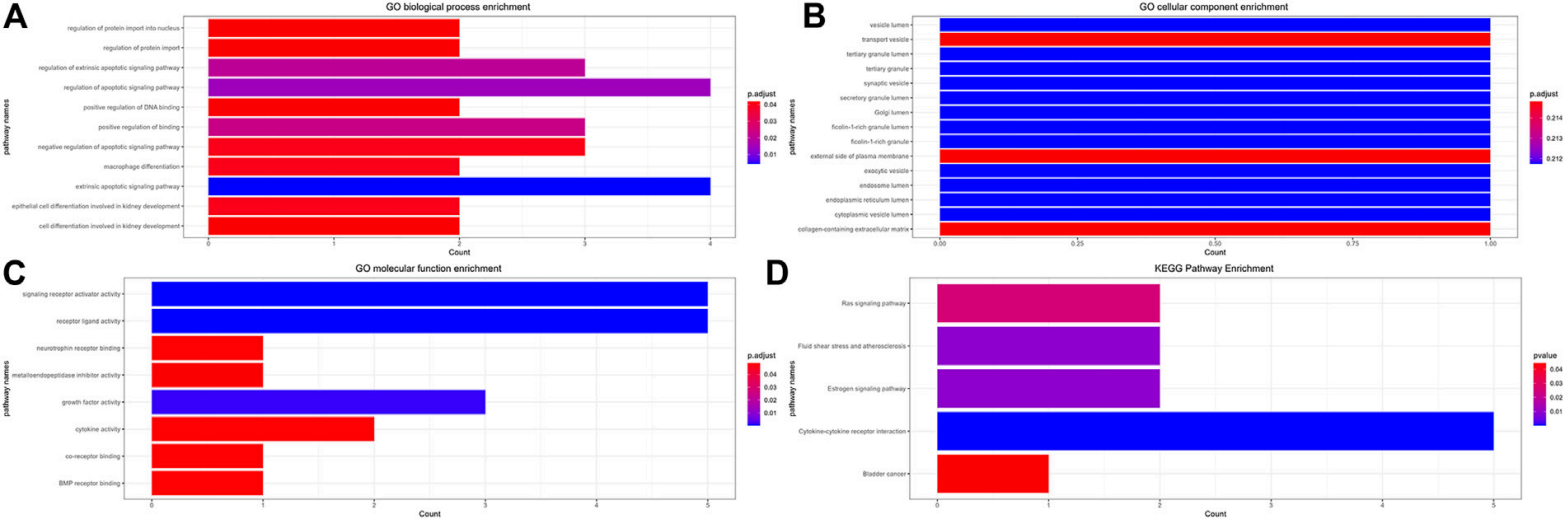
Functional GO and KEGG Enrichment Analysis of Immune Genes. **(A)** GO biological process enrichment, **(B)** GO cellular component enrichment, **(C)** GO molecular function enrichment, **(D)** KEGG pathway enrichment.

The GO and KEGG enrichment analysis results are shown in Table 4 and Figure 12. In the GO molecular function enrichment analysis, the differentially expressed immune genes were mainly enriched in receptor ligand activity, signaling receptor activator activity, growth factor activity, and other pathways. In biological process enrichment analysis, differential immune genes were mainly enriched in extrinsic apoptotic signaling pathway, regulation of apoptotic signaling pathway, regulation of extrinsic apoptotic signaling pathway, etc. In the cellular component enrichment analysis, the differential immune genes were mainly enriched in endosome lumen, tertiary granule lumen, and Golgi lumen, but the corrected *p* value was not statistically significant, suggesting that these pathways were not significant. In the KEGG enrichment analysis, the differential immune genes were mainly enriched in Cytokine-cytokine receptor interaction, Estrogen signaling pathway, Fluid shear stress and atherosclerosis. The immune genes mainly involved in enrichment analysis included NGF, IL32, BMP4, TNFRSF12A, and IL12RB2.

**TABLE 4:** Main results of GO and KEGG enrichment analysis for significantly expressed immune genes.

Type	Description	*p*	*p* adjust	Q value	gene ID	Count
GO_BP	extrinsic apoptotic signaling pathway	0.0000	0.004322	0.0021	NGF/BMP4/TNFRSF12A/PAK5	4
GO_BP	regulation of apoptotic signaling pathway	0.0000	0.014634	0.0071	MMP9/BMP4/TNFRSF12A/PAK5	4
GO_BP	regulation of extrinsic apoptotic signaling pathway	0.0001	0.020688	0.0100	BMP4/TNFRSF12A/PAK5	3
GO_MF	receptor ligand activity	0.0000	0.000115	0.0001	NGF/IL32/BMP4/OSGIN1/POMC	5
GO_MF	signaling receptor activator activity	0.0000	0.000115	0.0001	NGF/IL32/BMP4/OSGIN1/POMC	5
GO_MF	growth factor activity	0.0001	0.001442	0.0009	NGF/BMP4/OSGIN1	3
GO_CC	endosome lumen	0.0195	0.211736	0.1932	NGF	1
GO_CC	tertiary granule lumen	0.0305	0.211736	0.1932	MMP9	1
GO_CC	Golgi lumen	0.0570	0.211736	0.1932	NGF	1
KEGG	Cytokine-cytokine receptor interaction	0.0000	0.000317	0.0003	NGF/IL32/BMP4/TNFRSF12A/IL12RB2	5
KEGG	Estrogen signaling pathway	0.0095	0.150833	0.1419	MMP9/POMC	2
KEGG	Fluid shear stress and atherosclerosis	0.0096	0.150833	0.1419	MMP9/BMP4	2

### lncRNAs isolation, cDNA synthesis, and RT-qPCR

The expression levels of ADAMTS9-AS2, FAM167A-AS1, LINC01121, MIR124-2HG, MGC12916 and the internal reference RNA were detected by RT-qPCR using three of the above-mentioned cell lines. Compared with those lncRNAs in the non-responder group, expression levels of ADAMTS9- AS2, FAM167A-AS1, and MGC12916 lncRNA in the responder group were significantly up-regulated, while expression levels of LINC01121 and MIR124-2HG were down-regulated. The results are shown in Supplementary Figure S4 and Supplementary Table S6.

## Discussion

Many recent studies have focused on establishing the signatures of coding genes with or without non-coding RNAs to assess prognosis in patients with malignancies (Zhu et al., 2016; Qu et al., 2018; Hong et al., 2020). This research strategy divides prognostic groupings based on the absolute expression level of certain genes of interest, of which the simplicity of operation is an important advantage, as the prognostic model was set up on quantifying the expression levels of transcripts (Hong et al., 2020). However, the shortcomings are also obvious, because the expression of most genes varies greatly, especially for non-coding RNAs. This means that some basic experiments (such as rt-qPCR) should be used for practical verification to ensure that the genes of interest had sufficient expression (Zhu et al., 2021). To overcome the shortcomings of the above model, new prognostic models based on the strategy of the relative expression of immune-related gene pairing were proposed, which became a mainstream bioinformatics research method (Sun et al., 2020; Wu et al., 2020; Wang Y. et al., 2021). In this study, we were inspired by the new strategy and attempted to construct a reasonable model with ir-lncRNA pairs assess the prognostic value of ir-lncRNAs. Therefore, we did not apply rt-qPCR to verify their exact expression levels in the signatures in our study. To the best of our knowledge, there are no similar studies investigating the radioresistance of esophageal cancer.

First, we explored the expression level of ir-lncRNAs between non-responder ESCC patients and responder ESCC patients to investigate the potential mechanism of radioresistance in ESCC after chemoradiotherapy. We first screened 26 differentially expressed ir-lncRNAs from the GEO45670 dataset. The differential expression of these lncRNAs indicates that ir-lncRNAs had played an important role in the radioresistance of ESCC. Second, previous studies have shown that lncRNAs achieve biological functions through a variety of target genes, which may involve immune-related genes (Wang Y. et al., 2021). Therefore, we further explored the differential expression of immune genes, which were not only differentially expressed in esophageal cancer tissues with different response characteristics of radiotherapy, but also correlated with the expression of immune lncRNAs, and thus could be considered as target genes of ir-lncRNAs. Third, differential co-expression analyses were performed to classify ir-lncRNAs and immune genes, and the prognostic performance of ir-lncRNAs were validated *via* single-pairing approach along with a 0 or 1 matrix by cyclical calculation. Fourth, we performed univariate analysis combined with Lasso penalized regression to identify the significant irlncRNA pairs. Next, multivariate regression was used to identify ir-lncRNAs with independent effects. Fifth, the best model was set up by calculating AUC value with ROC, where the best cut-off point was applied to distinguish high- or low-risk groups of EPC patients. Sixth, we further evaluated this new model in a variety of clinical settings, including survival, clinical or pathological characters. At last, tumor-infiltrating immune cells between different radioresistance of esophageal cancer.

Radiotherapy is one of the main and effective treatments for advanced EPC, but the therapeutic outcomes of which are still unsatisfactory (Li et al., 2016; Yan et al., 2022), because the complete response rate of radiotherapy is less than 35%–40% (Mori et al., 2021). Radioresistance is the biggest problem and obstacle faced by radiotherapy in esophageal cancer, as poor response to radiotherapy could result in local failure after radiotherapy (Chen H. et al., 2020). Therefore, it is particularly important to search for radioresistance-related molecular markers and new radiosensitizers to enhance radio-sensitivity in ESCC cells and improve the survival of ESCC patients with radioresistance. Radioresistance is the focus and difficulty in cancer radiobiology research, which is also a clinically urgent issue (Wang et al., 2019; Yu et al., 2020; Sun et al., 2021; Liu et al., 2022). Genes are an intrinsic determinant of tumor radioresistance, and studies have shown that multiple genes can affect the radioresistance of esophageal cancer (Huang et al., 2019; Wang et al., 2019; Hua et al., 2020; Yu et al., 2020; Han et al., 2021; Sun et al., 2021; Liu et al., 2022). Practice has shown that it is common in clinical practice that even patients with esophageal cancer of the same stage and the same pathological type have great differences in the effect of radiotherapy after receiving the same radiotherapy regimen. The fundamental reason is the genetic differences between different individuals (Huang et al., 2019; Wang et al., 2019; Hua et al., 2020; Yu et al., 2020; Han et al., 2021; Sun et al., 2021; Liu et al., 2022). If the decisive genes of radioresistance can be screened out, it is of great significance for the study of radiosensitization, targeted therapy, and prediction of radiotherapy effect to guide individualized therapy. Although more molecular studies have been reported on the radioresistance or radiosensitivity of ESCC, the clinical significance of most molecular markers remains unclear and inconsistent due to the complexity and variability of detection methods.

Recent studies had found that a variety of lncRNAs can affect the radiosensitivity of EPC by regulating gene expression and key signal transduction pathways (Liu et al., 2021). Many lncRNAs are involved in the regulation of the tumor immune microenvironment, which are often referred to as immune-related lncRNAs (Huang et al., 2018). However, there is few reports on the relationship between ir-lncRNAs and radioresistance and prognosis of ESCC. Furthermore, the mechanism of ir-lncRNAs involved in the radioresistance is still unclear. In recent years, the use of bioinformatics methods for data mining at the molecular level provides new ideas for the study of molecular pathogenesis of various diseases including tumors (Zheng et al., 2020). Our present study used bioinformatics methods to re-analyze the radioresistance-related microarray data of esophageal cancer from GEO, and screened for differentially expressed ir-lncRNA genes. Through biological process annotation and signal pathway enrichment analysis, we have excavated some ir-lncRNA genes and immune-genes and potential signal pathways related to radioresistance to investigate the mechanism of radioresistance at the molecular level in ESCC. Zhu et al. (2021) had identified that a total of 111 immune-related lncRNAs were different expressed in ESCC, in which,14 lncRNAs markedly related to prognosis of ESCC were identified *via* univariate analysis. Finally, a model based on the 8-lncRNA signature was identified in the multiple regression model, which demonstrated that the 8-lncRNA signature has certain power in predicting the prognosis of ESCC patients (Zhu et al., 2021). Since the expression abundance of ir-lncRNAs is extremely low, which is different from the expression of protein genes, we used the relative expression status between different ir-lncRNAs to explore the biological functions of lncRNAs. Based on 26 differentially expressed ir-lncRNAs, we constructed 325 ir-lncRNA pairs in the GSE45670 dataset, and 180 of which were extracted to further study. As seen in our study, we found that there were differences in gene expression between non-responder ESCC and responder ESCC in terms of LINC01121, FAM167A-AS1, ADAMTS9-AS2, MGC12916, MIR124-2HG. ADAMTS9-AS2 and FAM167A-AS1 were up-regulated in the non-responder group, while LINC01121 and MIR124-2HG were down-regulated (*p* < 0.05). Among the five lncRNAs we identified; some studies have demonstrated their prognostic effects on malignant tumors. Shen et al. (2020) had found that a worse 5-year overall survival was detected in ESCC-patients with low-expressed ADAMTS9-AS2. Similar results were seen in clear cell renal cell carcinoma and bladder cancer (Song et al., 2019; Zhang et al., 2020), which indicates that ADAMTS9-AS2 is a tumor suppressor gene. However, the opposite results were found in patients with tongue squamous cell carcinoma, where high-expression of LncRNA ADAMTS9-AS2 promotes proliferation, migration and epithelial-mesenchymal transition (EMT) with poor prognosis, and low-expression was detected in patient with lymph node metastasis (Li Y. et al., 2019). In our study, the expression of ADAMTS9-AS2 was increased in non-responsive EPC patients, suggesting that ADAMTS9-AS2 was involved in the radioresistance of EPC, but the specific mechanism has not been reported. Some scholars have found that ADAMTS9-AS2 have been shown to play essential roles in temozolomide (TMZ) resistance in glioblastoma (GBM) (Yan et al., 2019). It should be mentioned that, although multiple lncRNAs, such as HOTAIR (Lv et al., 2013), CCAT2 (Zhang et al., 2015) and MALAT1 (Deng et al., 2016), have shown potential prognostic value in ESCC, the role of immune-related lncRNA signatures in prognosis has not been elucidated in the literature.

A few studies had addressed the role of immune-related lncRNAs in survival and prognosis of ESCC. A previous study had established an immune gene-based prognostic model for ESCC and esophageal adenocarcinoma (EAC) (Fei et al., 2021). Prognosis-related immune-gene-based model based on BMP1, EGFR, S100A12, HLA-B, TNFSF18, IL1B, and MAPT had proved to be useful for prognosis in ESCC (Fei et al., 2021). To verify the relationship between ir-lncRNA pairs and survival from ESCC, we adopted the GES53625 dataset as validation cohort. We constructed a novel prognostic prediction model consisting of three ir-lncRNA pairs, including LINC01121|FAM167A-AS1, ADAMTS9-AS2|MGC12916, and MIR124-2HG|FAM167A-AS1, which involved five ir-lncRNAs. We further confirmed by Wilcoxon rank sum test that there were significant differences in the expression levels of the above ir-lncRNAs in different risk score groups in the GES53625 cohort. In addition, the prognostic value of the novel model was confirmed by the multivariate Cox analysis. The overall survival in the high-risk group was significantly worse than that in the low-risk group (*p* < 0.001). The 1-year, 2-year, and 3-year prediction performance of this risk-model was 0.666, 0.702, and 0.686, respectively. Univariate and multivariate Cox analysis confirmed that prognostic riskscores based on three ir-lncRNA pairs were important prognostic factors for ESCC. Univariate and multivariate COX regression analysis indicated that the three ir-lncRNA pairs were associated with the prognosis of ESCC, and in the high-risk group ADAMTS9-AS2, LINC01121, MGC12916, and MIR124-2HG was up-regulated, FAM167A-AS1 was up-regulated. All the three ir-lncRNA pairs were markers of poor prognosis with worse overall survival for ESCC. A previous research showed that ADAMTS9-AS2 is a prognostic biomarker correlated with immune infiltrates and predicted a poorer overall survival when it was low expressed in lung adenocarcinoma (Lin et al., 2021). Li W. et al. (2020) had identified the expression levels of eight lncRNAs to establish a signature for predicting the survival of patients with ESCC. Li Z. Y. et al. (2020) found that the expression of lncRNA Rpph1 in patients with EPC was significantly higher than that in healthy participants (*p* < 0.05), and was positively correlated with cancer tissues (r = 0.681, *p* < 0.05). *In vitro* experiments confirmed that silencing lncRNARpph1 could up-regulate radio-induced pro-apoptotic-related proteins such as Bax, down-regulate anti-apoptotic-related proteins such as Bcl-2, thereby increasing radiotherapy-induced apoptosis of EPC cells. In addition, silencing lncRNA Rpph1 can also improve the radiosensitivity of EPC cells by reducing radiation-induced G2/M phase arrest and epithelial-mesenchymal transition (EMT) (Li Z. Y. et al., 2020). Wang et al. found that lncRNA CCAT2 was highly expressed in EPC cells, which can negatively regulate the expression of miR-145 and inhibit the phosphorylation of Akt, ERK, and p70 s6K1 to increase radioresistance. *In vitro* experiments confirmed that knockout of lncRNA CCAT2 can significantly increase radiation-induced apoptosis, thereby increasing radiosensitivity in EPC (Wang et al., 2020). In addition to mediating radioresistance, some lncRNAs also have radiosensitizing effects. Lin et al. reported that compared with the radioresistant ESCC cell line TE-1-R, the expression of lncRNA GAS5, and RECK was higher in the radiosensitive cell line TE-1, while the expression of miR-21 was lower in cell line TE-1 (Lin et al., 2020). Further research found that up-regulation of lncRNA GAS5 can increase the expression of RECK by inhibiting miR-21, reduce the viability and colony formation ability of EPC cells under radiation exposure, and increase radiation-induced apoptosis of cancer cells (Lin et al., 2020). All these evidences had suggested that lncRNAs play an important role in radioresistance or radiosensitivity of ESCC. In general, high-abundance lncRNAs have significant biological functions, especially lncRNAs with significant differences in expression (Yan et al., 2021). Different from the above studies, we focused on the ir-lncRNAs related to the immune environment or immune regulation of radioresistance. As expected, we found that ir-lncRNAs are involved in the bidirectional regulation of radiosensitivity, that is, some lncRNAs could promote radioresistance, while others may increase radiosensitivity.

In recent years, the study of the tumor immune microenvironment has taken a leading role in field of cancer research (Wang et al., 2022). The differential expression of immune genes is an important molecular mechanism leading to changes in the tumor immune microenvironment (Anderson, 2020; Peña-Romero and Orenes-Piñero, 2022). Several previous studies reported the prognostic value of a single immune-related gene in EPC or lung cancer, such as FGFR1, TNFRSF10B, and IL1B (Schabath et al., 2013; Takase et al., 2016; Zhang Y. et al., 2021). However, the study focused on the prognostic role of the ir-lncRNAs with microenvironment and immune cells in ESCC is lacking, especially for ESCC with radioresistance. Identification of prognostic value of the tumor microenvironment in esophageal cancer is necessary (Tan et al., 2021). In our study, we identified 11 immune genes with differential expression in the GSE 45670 cohort, and our findings further confirmed the involvement of immune alterations in the biological process of radiation resistance in ESCC. To further explore the changes and biological pathways in the immune microenvironment between patients in the non-responder group and patients in the responder group, CIBERSORT was carried out in this study as well, which was an analytical tool developed by Newman et al. (2015) to provide an estimation of the abundances of member cell types in a mixed cell population *via* gene expression data, and provided the possibility of identifying immune biomarkers for diagnosis and prognosis. The GSE 45670 dataset was utilized to evaluate immunity infiltration *via* the CIBERSORT algorithm. Growing evidence pointed to the underlying mechanisms by which the local immune microenvironment and immune cells drive tumorigenesis in many cancers (Greten and Grivennikov, 2019; Wang Q. et al., 2021; Kumar et al., 2022). Previous studies have shown that DNA damage repair-related genes, apoptosis-related genes, cellular hypoxia-related genes, cell cycle-related genes, and autophagy genes play important roles in radiosensitivity by changing the microenvironment (Chen et al., 2017; Tang et al., 2018; Zhang H. et al., 2021). Tumor-infiltrating immune cells (TIICs) in esophageal cancer tissue may be an important determinant of prognosis and therapy response (Lu et al., 2020). In our study, we found that there were some differences in infiltrating immune cells between the non-responder group and the responder group, which mainly manifested in differences in infiltration of activated mast cell and macrophage. Mast cells are an important member of innate immune cells. Circulating mast cells contribute to the growth and metastasis of many tumors, while mast cell infiltration in tumor tissue is closely related to tumor survival in some cancer (Welsh et al., 2005). In a study of lung cancer, it has been shown that more human mast cells infiltrated in cancer tissue improved the survival of cancer, suggesting that mast cells can participate in the antitumor immune process (Welsh et al., 2005). In ESCC cases with tumor complete remission, the number of mast cells infiltrated was higher, indicating that mast cells were involved in the therapeutic effect of radiotherapy.

There are a large number of tumor-associated macrophages (TAMs) in the tumor microenvironment, which have a high degree of interaction with tumor cells, tumor stem cells, epidermal cells, fibroblasts, T/B cells, and NK cells (Welsh et al., 2005). Although macrophages theoretically have the ability to destroy tumors, there is growing experimental evidence that TAMs promote tumor progression (Mantovani and Allavena, 2015). Resilience and diversity are two characteristics of macrophages, which means that macrophages have dual effects in tumor development (Cassetta and Pollard, 2020). According to the activation type of macrophages and their different roles in the tumor microenvironment, TAMs are generally classified into two functionally opposite subtypes, classically activated M1 macrophages and alternately activated M2 macrophages, both of which represent one of the main tumor-infiltrating immune cell types (Cassetta and Pollard, 2020). Basic research has found that a large number of TAMs proliferate after radiation, and at the same time release a large number of inflammatory signals (IL-1) and immunosuppressive signals (TGF-b). Unfortunately, the massive accumulation of macrophages can lead to tumor recurrence, which is very similar to TAMs-guided tissue damage repair after chemotherapy. M1 macrophages have antitumor effects, while M2 macrophages mainly play a role in promoting tumor growth, invasion and metastasis. Our study found that macrophages infiltrated more in esophageal cancer tissues in responder group, among which M0 macrophages were the main infiltrating cells. The infiltrating number of M2 macrophages was more often in the non-responder group, although there was no statistical difference compared with that in responder group. It is worth noting that both M1 and M2 macrophages have high degree of plasticity, which makes it possible to design appropriate methods to re-induce and re-educate them, thereby becoming an effective weapon against tumors. Different types of macrophages can be converted into each other upon tumor microenvironment changes or therapeutic interventions. In view of the important role of macrophages in tumor radioresistance, it needs to do in-depth research on the realization of their functions and their regulatory mechanisms, in order to find new anti-tumor targets.

Some limitations should be mentioned in our study. First, the prognostic model based on ir-lncRNA pairs was established through bioinformatics analyses from data available in the GEO databases. Hence, some further prospective trials or experimental data should be performed to validate the findings of this study. Second, our study found that most of clinical characters were not correlated with the riskscore, and tumor grade had a weak correlation with the riskscore. We speculate that preoperative adjuvant therapy altered gene expression status, resulting in clinical factors not correlated with gene expression. In addition, insufficient sample size may also be an important reason. Third, our study only initially explored the potential relationship between ir-lncRNAs risk signatures and immune cell infiltration, so further studies are needed to reveal the underlying mechanisms. Fourth, the effect of ir-lncRNAs on the immune microenvironment was indirectly speculated through immune genes, and there is currently a lack of direct evidence. More experiments are needed to confirm the impact of ir-lncRNAs on the radioresistance-related microenvironment of ESCC. Finally, as a preliminary exploratory study, it only has a qualitative role in the identification of ir-lncRNAs and the prognosis risk of ESCC patients. The relationship between ir-lncRNAs and the prognosis of ESCC patients has not been accurately quantified, which still needs to be further verified by multicenter studies with large samples.

In conclusion, ir-lncRNAs may be involved in the biological regulation of radioresistance in patients with ESCC. Changes in macrophage infiltration and immune gene expression are potential mechanisms of radiotherapy resistance, which are worthy of further study. This study had successfully established a prognostic risk model based on three ir-lncRNAs pairs, which is an important attempt to identify and predict the prognosis of ESCC. More importantly, it is a useful supplementary method to predict the prognosis of patients with esophageal squamous cell carcinoma based on TNM staging.

## Data Availability

The original contributions presented in the study are included in the article/Supplementary Material, further inquiries can be directed to the corresponding author.

## Supplementary material

Supplementary Figure S1 Selection of the optimal candidate genes in the LASSO model.

Supplementary Figure S2 LASSO coefficients of prognosis-associated ir-lncRNA pairs, each curve represents a gene.

Supplementary Figure S3 Differential distribution of risk score with different clinical characters.

Supplementary Figure S4 The expression levels of ir-lncRNAs detected by RT-qPCR, **p* < 0.05, ***p* < 0.01, ****p* < 0.001.
