# Supplementary figures and images for: A novel immune-related radioresistant lncRNAs signature based model for risk stratification and prognosis prediction in esophageal squamous cell carcinoma
Source: Front Genet. 2022 Sep 6;13:921902. doi: 10.3389/fgene.2022.921902 (PMC9485730; doi:10.3389/fgene.2022.921902)

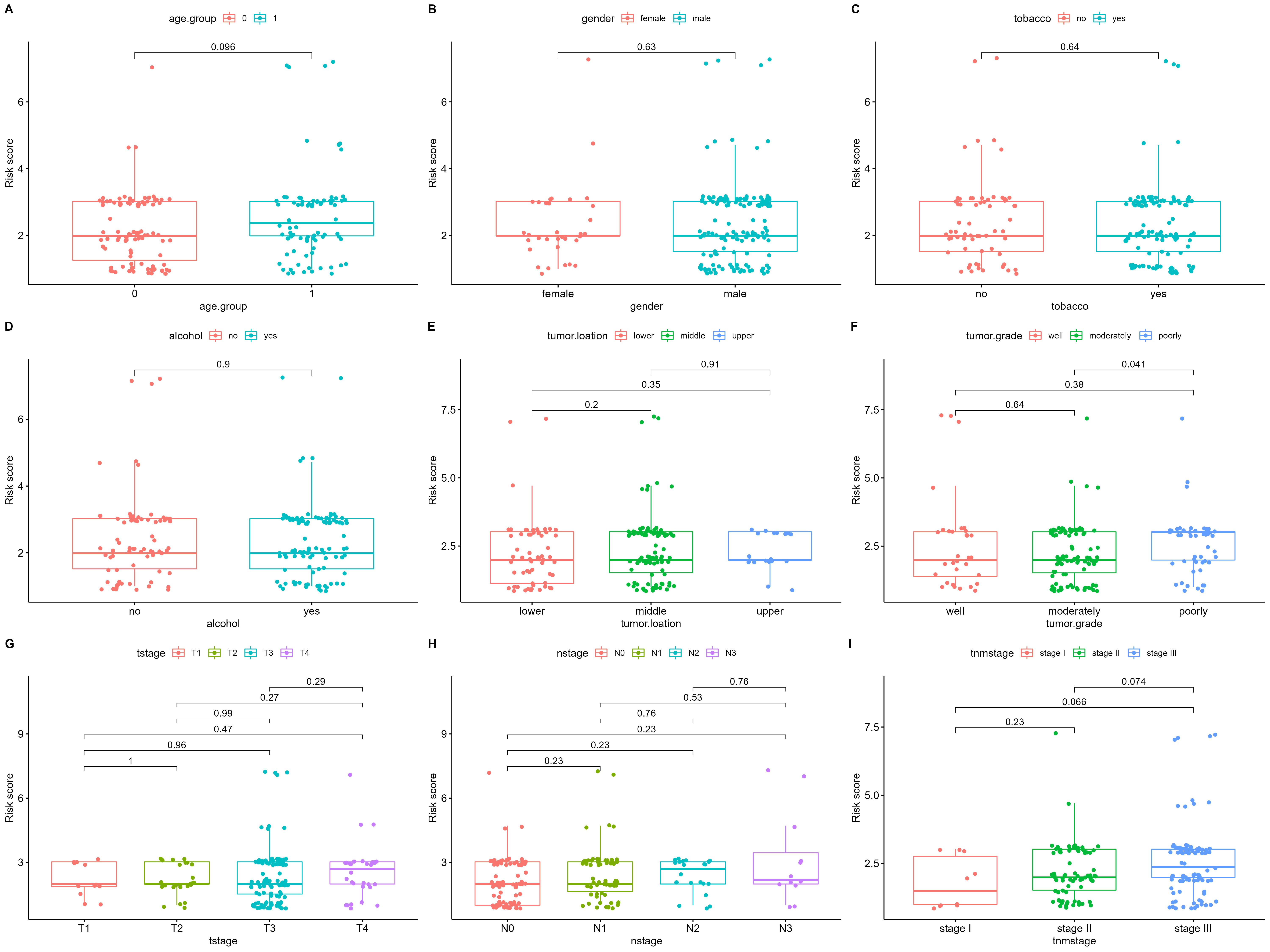

Supplement: Supplementary file 1 [file Image3.JPEG]

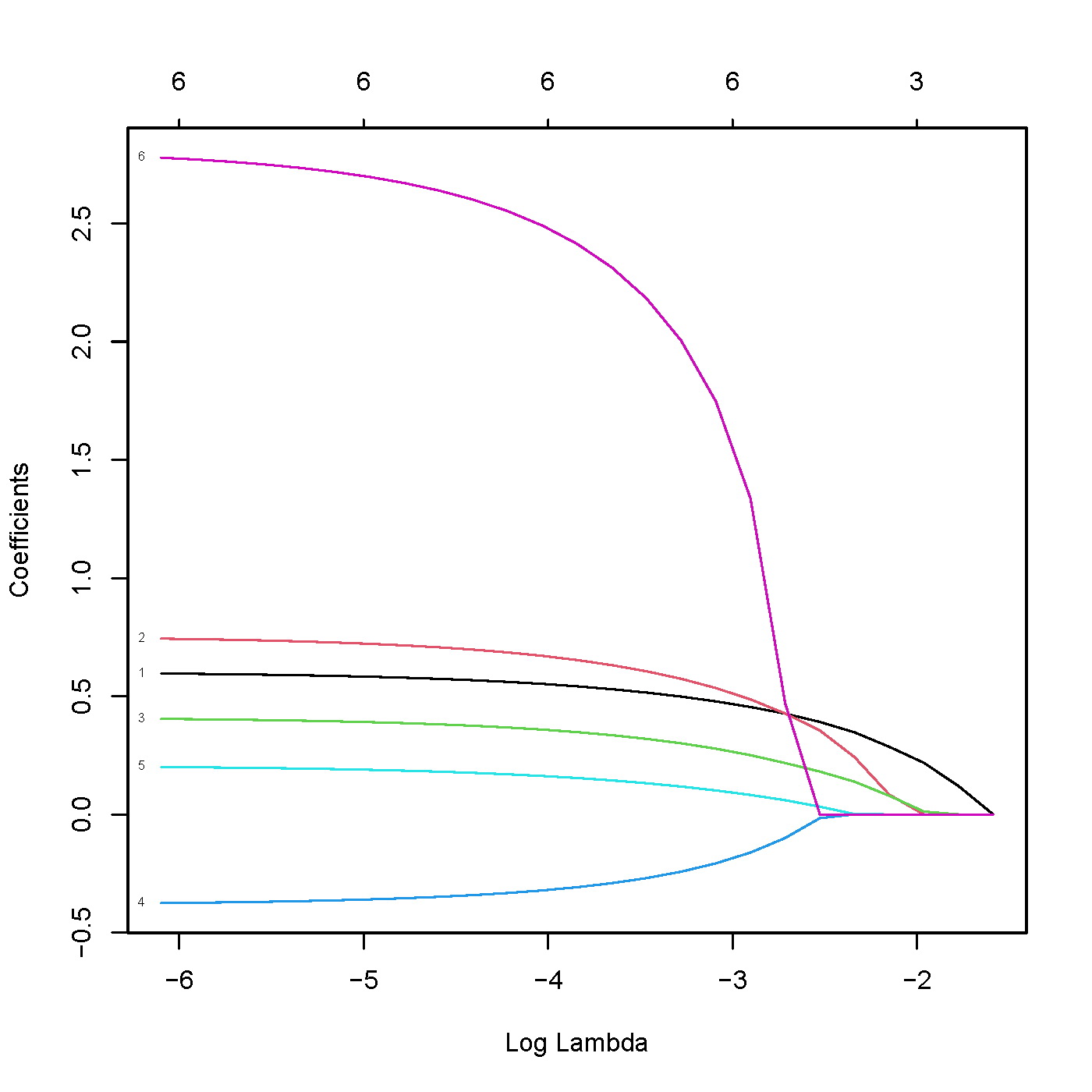

Supplement: Supplementary file 2 [file Image1.JPEG]

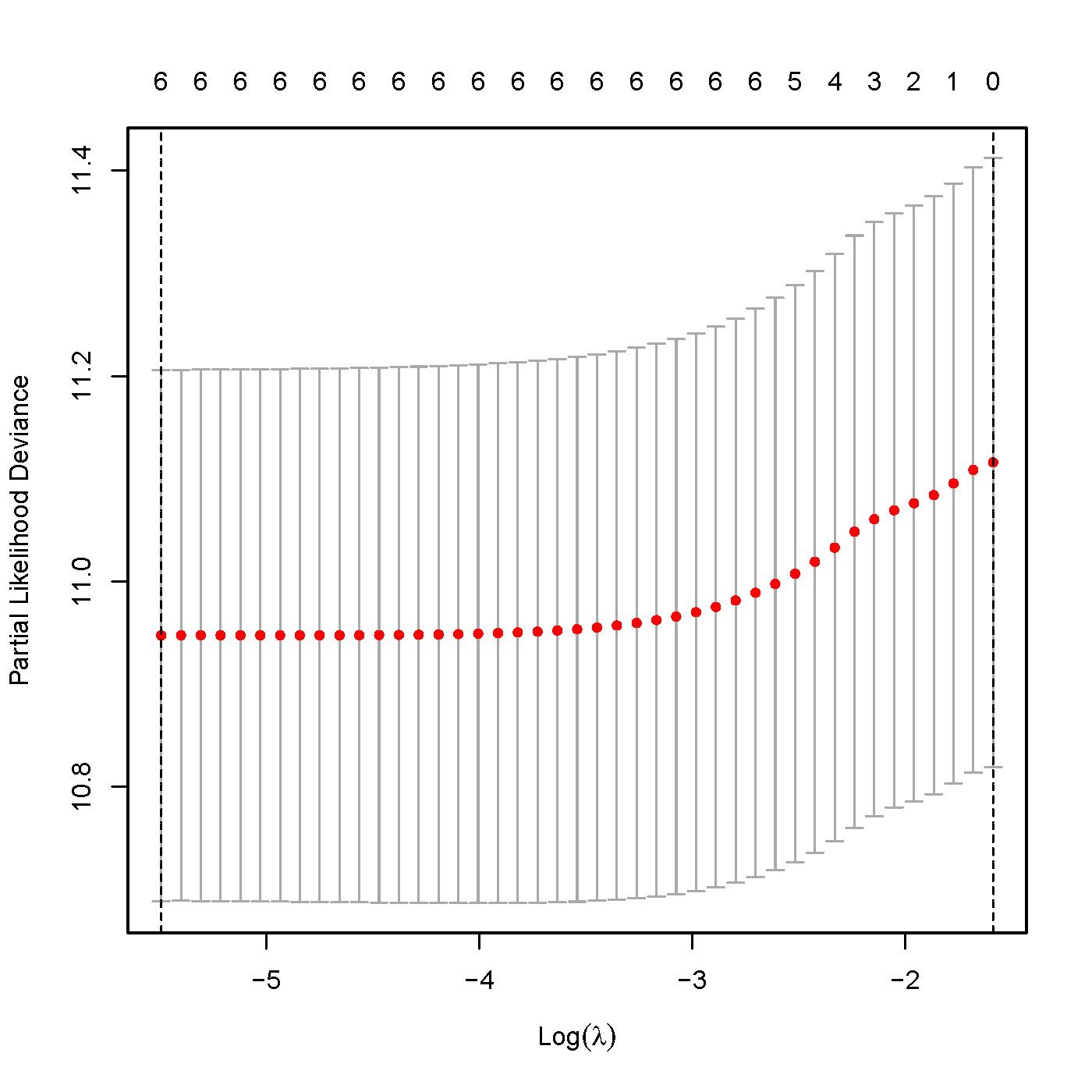

Supplement: Supplementary file 3 [file Image2.JPEG]

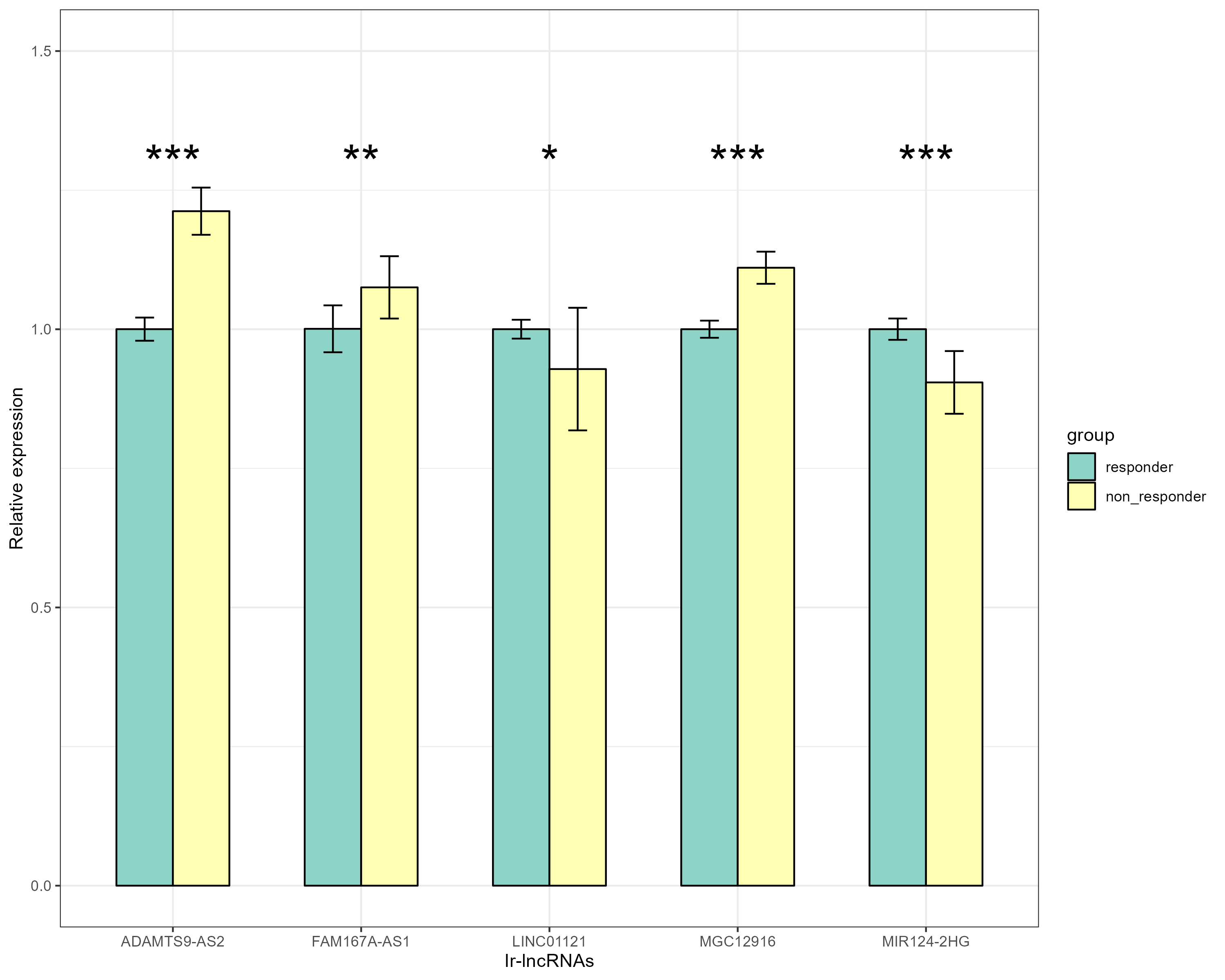

Supplement: Supplementary file 4 [file Image4.TIFF]
